# Assessment of the Frequency of Autoantibodies in Chronic Viral Hepatitis

**DOI:** 10.12669/pjms.311.6053

**Published:** 2015

**Authors:** Akif Acay, Kasim Demir, Gulsah Asik, Havva Tunay, Gursel Acarturk

**Affiliations:** 1 Akif Acay, Department of Internal Medicine, Afyon Kocatepe University, Faculty of Medicine, Afyonkarahisar, Turkey.; 2 Kasim Demir, Department of Gastroenterology, Afyon Kocatepe University, Faculty of Medicine, Afyonkarahisar, Turkey.; 3 Gulsah Asik, Department of Medical Microbiology, Afyon Kocatepe University, Faculty of Medicine, Afyonkarahisar, Turkey.; 4 Havva Tunay, Department of Infectious Diseases & Clinical Microbiology, Afyon Kocatepe University, Faculty of Medicine, Afyonkarahisar, Turkey.; 5 Gursel Acarturk, Department of Gastroenterology, Afyon Kocatepe University, Faculty of Medicine, Afyonkarahisar, Turkey.

**Keywords:** Chronic hepatitis B, Chronic hepatitis C, Autoimmune diseases, Autoantibodies

## Abstract

**Objective:**

To examine the occurrence frequency of auto-antibodies and autoimmune diseases in patients with chronic hepatitis B or C.

**Methods:**

A total of 67 patients diagnosed with chronic hepatitis B and 77 patients diagnosed with chronic hepatitis C infection based on HBs Ag, Anti HCV, HBe Ag, Anti HBe Ag, HBV DNA, HCV RNA, liver ultrasound, and liver biopsy results as well as 48 healthy individuals were included in this study. ANA, anti dsDNA, anti LKM, Anti-SMA, AMA, C-ANCA, P-ANCA, anti-SSA, anti-SSB, anti-Scl-70, anti Jo-1, anti-U1snRNP, anti-centromere, anti-Jo-1, anti tpo, and anti tg were studied in all individuals in each study group.

**Results:**

ANA positivity was detected in 8 (12%), 15 (19%) and 2 (4%) individuals in HBV, HCV and control groups, respectively. The difference between the groups was significant (P=0.04). Similarly, anti Tg was positive in one subject in HBV group, in 6 subjects (7%) in HCV group, and in one subject among controls, the difference being significant (P=0.04). There were no significant differences between the study groups in the frequency of other auto-antibodies.

**Conclusion:**

Similar to studies involving patients who received interferon and/or antiviral agents, an increased frequency of auto-antibodies was also detected in our patient group consisting of interferon and anti-viral naive subjects. The increase in the frequency of auto-antibodies reached statistical significance among individuals with HCV infection. Thus, pre-treatment assessment of auto-antibodies in newly diagnosed cases of chronic hepatitis B or hepatitis C infection may provide beneficial information on the future occurrence of auto-immune responses in these patients.

## INTRODUCTION

Auto-antibodies are produced against the antigens of an organism such as proteins, nucleic acids, carbohydrates, lipids, and multi molecular complexes. Auto-antibodies can be organ-specific or systemic. They have not only diagnostic and pathogenetic relevance, but also provide prognostic information. Commonly tested auto-antibodies include anti-nuclear antibodies (ANA), antibodies directed against DNA, antibodies directed against proteins that bind to nucleic acids (ENA), those directed against phospholipids, and anti-neutrophil cytoplasmic antibodies (ANCA). Additionally, anti-bodies specific to certain tissues or organs may also be produced by the immune system (e.g. those against hepatic, renal, gastric, intestinal, thyroid, pancreatic, muscular, testicular, dermatological, or neurological tissues). Viral, bacterial or parasitic infections may result in the development of auto-immune conditions due to their ability to induce robust inflammatory responses in a variety of organ systems. An infectious agent may induce a variety of different auto-immune diseases or different agents may play a role in the pathogenesis of a single auto-immune condition.^1^^,^^2^

Previous studies have clearly established the co-occurrence of certain immunological or extra-hepatic syndromes in patients with chronic hepatitis B virus (HBV) or hepatitis C virus (HCV) infection. This is particularly relevant for HCV infections.^3^^,^^4^ The most common auto-immune diseases associated with chronic HBV infection are membranous glomerulonephritis and systemic necrotizing vasculitis. Similarly an association between HCV infection and mixed cryoglobulinemia, auto-immune thyroiditis, nephropathy, sicca syndrome, idiopathic pulmonary fibrosis, lichen planus, type II diabetes mellitus, chronic polyarthritis, and porphyria cutanea tarda has been shown.^5^^-^^9^

In both group of patients, the most important risk factors for the development of auto-antibodies include age, female gender, and the extent of liver fibrosis. Although clinically rare, these extra-hepatic conditions may lead to a significant increase in morbidity and mortality. Auto-antibodies have been detected in nearly 20% of patients with HCV infection. Such antibodies are present in patients with auto-immune hepatitis and their frequency in subjects with chronic viral hepatitis bears important clinical implications due to the clear evidence suggesting an adverse effect of interferon treatment on the clinical course of auto-immune hepatitis.^10^^,^^11^ Therefore, in the present study our objective was to examine the frequency of auto-antibodies among patients with chronic hepatitis B or C virus infection.

## METHODS

Patient records of 170 individuals (91 cases of chronic hepatitis C, 79 cases of chronic hepatitis B) followed up at the chronic hepatitis outpatient unit of Afyon Kocatepe University Hospital between August 2012 and December 2013 were retrieved from the hospital database. A telephone call invitation was made to all patients. Demographic data such as age and gender were recorded, detailed medical history was obtained, and physical examination was performed routinely in each patient. The following laboratory tests were performed: anti-HCV, HCV RNA, anti-HBs, HBsAg, Anti-HBe, HBV DNA, serum alanine amino transferase (ALT), aspartate amino transferase (AST), prothrombin time (PTT), total protein and albumin. Also liver ultrasound was performed. All patients with chronic hepatitis B and only the patients with chronic hepatitis C, suspected to be in pre-cirrhotic condition underwent liver biopsy. The ones with hepatitis C did so in order to exclude liver cirrhosis. 

Patients with a previous history of interferon or anti-viral treatment, negative HCV RNA or HBV DNA result, liver cirrhosis, or hepatocellular carcinoma were excluded from the study. The final study population (n=144) consisted of 77 patients with hepatitis C, and 67 patients with hepatitis B. All individuals in the control group (n=48) had negative HBs Ag and anti-HCV. Thus, three study groups were defined: i.e. chronic hepatitis B, chronic hepatitis C, and control.

The following tests were performed in all study participants for the study purposes: anti-nuclear antibody (ANA),  anti-double stranded DNA (anti-dsDNA), anti-liver/kidney microsomal type 1 (LKM-1), anti-smooth muscle antibody (Anti-SMA), antimitochondrial antibodies (AMA), perinuclear antineutrophil cytoplasmic antibody (p-ANCA), cytoplasmic ANCA (c-ANCA), anti-SSA/Ro, anti-SSB/La, anti-Scl-70, anti-centromere antibody, Anti-Jo-1, anti-thyroid peroxidase (anti-tpo), Anti thyroglobulin (anti-Tg), Anti-U1small nuclear (sn) RNP (Anti-U1snRNP), Anti Smith antibody (Anti-Sm) and anti-cardiolipin antibodies (ACA). ELISA (Vitros ECiO, Ortho Clinical Diagnostics) was used to examine hepatitis markers (HBs Ag, Anti HCV, HBe Ag, Anti HBe Ag); and quantitative Real Time Polymerase Chain Reaction (PCR) was used to detect HCV-RNA (Cobas Taqman48, Roche) at the ELISA and Molecular Microbiology Unit, Microbiology Laboratory, Medical Faculty of Afyon Kocatepe University. 

IFA method was utilized for the detection and pattern examination of ANA (ANA profile 3, Euroimmun) using fixed HEp-2 cells as substrate. In line with the manufacturer's instructions, the patient sera were diluted at 1:100 for the test procedures. For patients with ANA positivity at a dilution of 1:100, the test was repeated at 1:200, and 1:400 and at further dilutions, when required. Anti-dsDNA, p-ANCA, c-ANCA and Anti-SMA were tested using commercial Euroimmun kits based on Indirect Fluorescein Antibody method (IFA) method, according to manufacturer's instructions at the Serology-Immunology Unit, Medical Faculty of Afyon Kocatepe University*.*

*Anticardiolipin antibody* was tested by enzyme-linked immunosorbent assay (ELISA) for cardiolipin. Also Anti-ENA (Extractable Nuclear Antigen) assay was performed with commercial kits (Euroline ANA Profile 1, Euroimmun) based on Immunblotting method, with each strip containing nRNP/Sm (U1-nRNP), Sm, SS-A, Recombinant Ro-52 (Ro-52, 52kDa), SS-B, DNA-Topoisomerase I (Scl-70), PM-Scl, Histidyl-tRNA synthetase (Jo-1), Centromer protein B (CENP B), Double stranded DNA (dsDNA), and Nucleosome, Histone antigens. Anti-LKM, AMA-M2 (Euroline Liver Profile, Euroimmun,) Anti-Tg and Anti-TPO (Euroassay AntiTg+TPO IgG, Euroimmun) tests were performed using the dot blot method at the Serology-Immunology unit.

***Statistical analyses:*** Statistical analyses were performed using SPSS V. 18.0. The variables were investigated using visual (histograms, probability plots) and analytical methods (Kolmogorov Smirnov test) to determine the normality of distributions. The results were expressed as mean ± standard deviation and median value (min-max range). ANOVA was used to compare parameters with normal distribution among study groups (chronic hepatitis B, chronic hepatitis C, and control). Data was analyzed by use of Chi-square test and Fisher’s exact test was performed to test for differences in proportions of categorical variables between two or more groups. The level of p<0.05 is considered significant.

## RESULTS

The mean age of subjects with hepatitis B infection, hepatitis C infection, and controls were 44.55±12.3, 45.38±10.98 and 43.21±7.3 years, respectively. The number of females and males in HBV, HCV and control groups were 29 and 38, 37 and 40, and 20 and 28, respectively. There were no significant differences between the study groups in terms of age and gender (P=0.49, P=0.75, respectively).

ANA positivity was detected in 8 (12%), 15 (19%) and 2 (4%) individuals in HBV, HCV and control groups, respectively. This difference between the groups was significant **(P=0.02).** Similarly, anti-Tg was positive in one subject in HBV group, in 6 subjects (7%) in HCV group, and in one subject among controls, again the difference being significant (**P=0.03**). There were no significant differences between the study groups in the frequency of other auto-antibodies. The demographic characteristics and laboratory findings in study groups is summarized in Table-I.

Between-group comparisons with regard to ANA showed a significant differences in the number of patients with ANA positivity between the HCV group (15 patients, 19%) and controls (2 subjects, 4%) (p=0.01). However, the difference between HBV and HCV and HBV and control groups were not statistically significant (p=0.22 and p=0.14, respectively) Overall, there were 25 ANA positive patients in the study, 19 being females and 6 being males. Table 2 depicts the gender and group distribution of individuals with ANA positivity.

## DISCUSSION

Numerous studies have reported an increased incidence of auto-antibodies and auto-immune disorders in subjects with chronic hepatitis B or C infection. However, in contrast with these previous studies .the prevalence of auto-antibodies and auto-immune disorders were examined in the present study in a group of interferon and anti-viral naive patients with HBV DNA and HCV RNA positivity.

Of the study subjects with chronic hepatitis C infection, 19% had ANA positivity. Also 2 patients had ASMA, one patient had anti-SS-A, one patient had AMA, and one patient had anti-LKM positivity (all were also ANA positive). However, no autoimmune disorders were detected in these patients. Additionally, 6 patients had anti-TG and two had anti-TPO positivity, with no abnormality in thyroid function tests. ANA was positive in 12% of the patients with chronic hepatitis B infection. Also one patient had anti-dsDNA, one had P-ANCA, and two had anti-TPO and anti-TG positivity, with normal thyroid function tests no auto-immune diseases. Similar to our findings, in a study by Clifford et al.^12^ involving a total of 117 patients with chronic hepatitis C infection, 14%, 2%, and 66% of the patients had ANA, AMA, and ASMA positivity, respectively. In another study by Floreani et al.^13^ 9.8%, 11%, and 5% of the HCV patients had ANA, ASMA, and AMA plus ASMA positivity, respectively. Lenzi et al.^14^ Compared the incidence of non-organ specific antibodies in hepatitis C and hepatitis B positive individuals. The frequency of ANA, ASMA, and AMA positivity were compared in 226 cases with anti-HCV positivity, 87 cases with HBsAg positivity, and in 226 healthy controls. Results showed an antibody positivity of 25%, 6%, and 7% among HCV, HBV, and control subjects, respectively. In a Turkish study by Bayraktar et al.^15^ involving 162 patients with chronic hepatitis C infection and 41 patients with auto-immune chronic hepatitis, the ANA, AMA, and ASMA positivity rates in chronic hepatitis C patients were 63%, 4%, and 65%, respectively. In that study, the reported rates of positivity were remarkably higher compared to those observed in our study, particularly in the group of patients with chronic hepatitis C infection. We believe that the difference between study results might reflect the exclusion of patients with previous use of anti-viral agents and interferon from our study.

Also, several authors have suggested that the progressive HCV infection may increase the frequency of anti-cardiolipin antibodies (ACA) and that it may lead to an increase in cell surface phospholipids and pro-inflammatory cytokines responsible for the production of ACA, thus resulting in endothelial and liver damage.^16^ Sahan et al.^17^ used liver biopsy to assess the hepatitis activity index in a total of 25 subjects with chronic hepatitis C infection and measured anti-cardiolipin antibody immunoglobulin M (ACA IgM). Of the overall group, 20% had ACA IgM positivity, with a significant correlation between ACA IgM and elevated hepatitis activity index. But, we found that ACA was positive in two patients in HBV group, in 6 patients (7%) in HCV group.

In our study, despite an increased frequency of auto-antibodies among patients with HBV or HCV infection, no patients were found to have auto-immune disorders. A previous study by Mert et al.^18^ provided supportive data for our findings where prevalence of anti-HCV in patients with auto-immune disorders and auto-antibodies in chronic hepatitis C patients were explored. In that study involving a total of 81 individuals, 35 patients had chronic hepatitis C infection, 27 had auto-immune endocrine disorders (16 with Hashimoto's disease, 8 with Graves’ disease, 2 with Addison's disease, and 1 auto-immune hypoparathyroiditis) and 19 had Sjögren syndrome. While 23%, 36%, 4.5%, 6%, and 6% of the patients with chronic hepatitis C infection had ANA, ASMA, anti-LKM-1, thyroid antibody, and AMA positivity, respectively, none of the subjects with auto-immune disorders had anti-HCV positivity. These results point out to the possibility that HCV infection may actually be devoid of the potential to cause auto-immude diseases. We believe that the future research should be directed to clarify this possibility.

***Limitations:*** The major limitation of this study is its cross-sectional nature. In addition, since our study was not designed prospectively, we may not have been able to detect the autoimmune diseases to emerge later on. Another significant limitation is the lower number of the subjects in the control group compared to chronic hepatitis B, and chronic hepatitis C groups, and there was also lower number of patients who were positive for autoantibodies.

**Table-I:** The demographic characteristics & laboratory findings in study groups

	Chronic Hepatitis B n: 67	Chronic Hepatitis C n: 77	Control group n: 48	P
Age	44.55±12.3	45.38±10.98	43.21±7.3	0.49*
Gender (F/M)	29/38	37/40	20/28	0.75
ANA	8(%12)	15(%19)	2(%4)	0.02
Anti-dsDNA	1(%12)	-	-	0.45
LKM-1	-	1(%1.3)	-	0.36
Anti-SMA	-	2(%2.6)	-	0.25
AMA	-	1(%1.3)	-	0.55
C-ANCA	-	-	-	-
P-ANCA	1(%1.5)	-	-	0.24
Anti-SSA/Ro	-	1(%1.3)	-	0.5
Anti-SSB/La	-	-	-	-
Anti-Scl-70	-	-	-	-
Anti-centromere	-	-	-	-
Anti-Jo-1	-	-	-	-
Anti-tpo	2(%3)	2(%2.6)	1(%2)	0.78
Anti-Tg	1(%1.5)	6(%7)	1(%2)	0.03
Anti-U1snRNP	-	-	-	-
Anti-Sm	-	-	-	-

Chi-square test and Fisher’s exact test were performed.

**ANA:** Anti-nuclear antibody, **Anti-ds DNA**: Anti-double stranded DNA, **LKM-1**:

Anti-liver/kidney microsomal type 1, **Anti-SMA**: Anti-smooth muscle antibody;

**AMA**: Antimitochondrial antibodies, **c-ANCA**: Cytoplasmic ANCA, **p-ANCA**:

Perinuclear antineutrophil cytoplasmic antibody, **Anti-SSA/Ro, Anti-SSB/La**, **Anti-Scl-70**,

A**nti-centromere antibody, Anti-Jo-1, Anti-tpo**: Anti- thyroid peroxidase, **Anti-Tg**:

Anti thyroglobulin, **Anti-U1snRNP**: Anti-U1small nuclear (sn) RNP and

**Anti-Sm**: Anti Smith antibody.

* : ANOVA was used to compare the age distribution among the groups.

**Table-II:** The gender and group distribution of individuals with ANA positivity

	**ANA Positive**	
	Female	Male
Chronic Hepatitis B	6	2
Chronic Hepatitis C	11	4
Control Group	2	-

**ANA: **Anti-nuclear antibody.

In conclusion, similar to studies involving patients who received interferon and/or antiviral agents, an increased frequency of auto-antibodies was also detected in our patient group consisting of interferon and anti-viral naive subjects. The increase in the frequency of auto-antibodies reached statistical significance among individuals with HCV infection. Also, although statistically not significant, an increase in the frequency of auto-antibodies was seen in those with HBV infection. In this regard, it may be reasonable to test for auto-antibodies in newly diagnosed patients before commencing treatment. We acknowledge some limitations to our analysis. 

## Authors Contribution:

**Akif Acay. S**tudy design and conception and writing of the manuscript. 

**Kasim Demir.** Interpretation of data and writing of the manuscript.

**Gulsah Asik. **Data acquisition and analysis.

**Havva Tunay** drafted the manuscript and reviewed it critically for important intellectual content and reviewed the manuscript critically and provided final approval of the version to be published. 

**Gursel Acarturk **is the guarantor of this work and, as such, had full access to all the data in the study and takes responsibility for the integrity of the data and the accuracy of the data analysis.
